# Blocked *O*-GlcNAc cycling disrupts mouse hematopoeitic stem cell maintenance and early T cell development

**DOI:** 10.1038/s41598-019-48991-8

**Published:** 2019-08-29

**Authors:** Lara K. Abramowitz, Christelle Harly, Arundhoti Das, Avinash Bhandoola, John A. Hanover

**Affiliations:** 1 Laboratory of Cellular and Molecular Biology, National Institute of Diabetes and Digestive and Kidney Diseases, National Institute of Health, Bethesda, MD 20892 USA; 2 Laboratory of Genome Integrity, Center for Cancer Research, National Cancer Institute, National Institutes of Health, Bethesda, MD 20892 USA; 3 CRCINA, INSERM, CNRS, Université d’Angers, Université de Nantes, Nantes, France; 4 LabEx IGO ‘Immunotherapy, Graft, Oncology’, Nantes, France

**Keywords:** Lymphopoiesis, Haematopoietic stem cells

## Abstract

Small numbers of hematopoietic stem cells (HSCs) balance self-renewal and differentiation to produce the diversity and abundance of cell types that make up the blood system. How nutrients are recruited to support this massive differentiation and proliferation process remains largely unknown. The unique metabolism of adult HSCs, which rely on glycolysis and glutaminolysis, suggests a potential role for the post-translational modification *O*-GlcNAc as a critical nutrient signal in these cells. Glutamine, glucose, and other metabolites drive the hexosamine biosynthetic pathway (HBP) ultimately leading to the *O*-GlcNAc modification of critical intracellular targets. Here, we used a conditional targeted genetic deletion of the enzyme that removes *O*-GlcNAc, *O*-GlcNAcase (OGA), to determine the consequences of blocked *O*-GlcNAc cycling on HSCs. *Oga* deletion in mouse HSCs resulted in greatly diminished progenitor pools, impaired stem cell self-renewal and nearly complete loss of competitive repopulation capacity. Further, early T cell specification was particularly sensitive to *Oga* deletion. Loss of *Oga* resulted in a doubling of apoptotic cells within the bone marrow and transcriptional deregulation of key genes involved in adult stem cell maintenance and lineage specification. These findings suggest that *O*-GlcNAc cycling plays a critical role in supporting HSC homeostasis and early thymocyte development.

## Introduction

The mammalian hematopoietic system is comprised of diverse cell types that each have unique functions. There are erythrocytes that carry oxygen, lymphocytes that are the cornerstones of adaptive immunity and myeloid cells, that have unique roles ranging from innate immunity to blood clotting. As abundant and diverse as these cells are, they all originate from a limited pool of hematopoietic stem cells (HSCs) residing in the bone marrow. HSCs are required to maintain homeostasis despite constant insults, like bleeding or infection. To do this, HSCs must strike a balance between quiescence to maintain HSC pools and proliferation and differentiation. One way that HSCs regulate this balance is through metabolic reprogramming, in fact, HSCs rely heavily on glycolysis and glutaminolysis to meet their unique energy needs^1,2^. However, what regulates how nutrients are recruited and support HSC homeostasis remain largely unknown. Here, we provide evidence that cycling of the nutrient-sensitive post-translational modification *O*-GlcNAc plays a key role in this process.

Glucose, glutamine, and other nutrient-derived metabolites help fuel the hexosamine biosynthetic pathway (HBP), ultimately contributing to multiple glycan classes including the post-translational modification *O*-GlcNAc. *O*-GlcNAc is a dynamic modification attached to serines and threonines of thousands of intracellular proteins^3^. Added to proteins by *O*-GlcNAc transferase (OGT) and removed by *O*-GlcNAcase (OGA), *O*-GlcNAc cycling integrates nutrient availability with molecular processes like transcription, translation, proteostasis and signaling^4^. Because it plays diverse roles in cell physiology, *O*-GlcNAcylation is poised to influence cells reliant on glycolysis and glutaminolysis like HSCs and T cells. In fact, *Ogt* is essential for embryonic stem cell (ESC) maintenance, differentiation and lineage specification^5–7^. *O*-GlcNAc cycling has been linked to polycomb repression^8–11^ and trithorax activation^8,12,13^ which act to define and shape early cell fate decisions. Many of the core components of the pluripotency network, including the Yamanaka factors OCT4, SOX2 and cMYC^6,14^ have been identified as *O*-GlcNAc modified. Indeed, mouse deletion models have shown that the enzymes of *O*-GlcNAc cycling are vital for early development, with *Ogt* deletion being embryonic lethal^5,15^, and *Oga* deletion perinatal lethal^16^. Further, a recent report suggested *O*-GlcNAc cycling influences *in vitro* erythrocyte differentiation^17^.

Downstream of HSCs, the T cell lineage also utilizes glucose and glutamine throughout development in the thymus. In fact, dynamic increases in protein *O*-GlcNAc levels has been defined at key stages of T cell development^18^. Dynamic *O*-GlcNAcylation has been shown to be essential for generation of mature CD4+ and CD8+ T cells^15,18^. Although *O*-GlcNAc addition is required for thymic development^15,18^, it remains unclear how blocked *O*-GlcNAc turnover by selective loss of *Oga* might impact early thymocytes. Moreover, the role of dynamic *O*-GlcNAcylation had not been investigated in earlier hematopoietic cell populations and hematopoiesis. Because *O*-GlcNAc cycling is a crucial feature in cell populations reliant on glycolysis and glutaminolysis, we anticipated that stable *O*-GlcNAcylation would disrupt HSC homeostasis. In fact, *O*-GlcNAc has been shown to regulate key factors in HSC maintenance like HIF1α^19^ and cMYC^14,18^. Here, we investigated the consequences of blocking *O*-GlcNAc turnover on HSC homeostasis and T cell development. We found that mice in which *Oga* had been selectively deleted in HSCs had depleted HSC and progenitor populations with diminished self-renewal and competitive repopulation capacity. Loss of *Oga* resulted in an increase in apoptosis and transcriptional analysis revealed that nutrient transport and FGF signaling likely contributed to HSC dysfunction in mutant HSCs. Our data suggested that the processes of *O*-GlcNAc addition and removal each play a key part in regulating critical steps of hematopoiesis. These findings have important implications for the role of hexosamine signaling in the maintenance of stem cell populations giving rise to immune homeostasis.

## Results

### Tissue specific Oga deletion resulted in diminished HSC and progenitor pools

**Figure 1:**
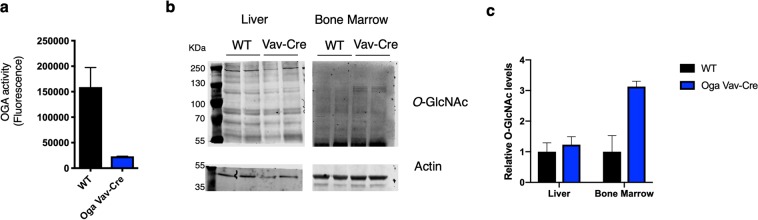
Deletion of *Oga* in hematopoietic stem cells. (**a**) OGA activity was quantified with an established *in vitro* fluorescence assay^21^ using lysates from wildtype (WT) or *Oga*^*Vav*-*Cre*^ bone marrow, N = 3, error bars represent standard deviation. (**b**) *O*-GlcNAc was assessed by western blot using lysates from liver and bone marrow of WT and *Oga*^*Vav*-*Cre*^ (Vav-Cre) mice using RL2 for *O*-GlcNAc and β-Actin as loading control. Lysates from liver and bone marrow were run out on separate gels and transferred to nitrocellulose membranes (full length blots can be found in Supplemental Fig. S7) that were cut at 55 kDa to allow for simultaneous quantitation of *O*-GlcNAc and β-Actin. (**c**) Quantitation of western blot from panel b with O-GlcNAc intensity normalized to β-Actin intensity, with error bars representing range. Black bars represent wildtype and blue bars represent *Oga*^*Vav*-*Cre*^.

Previous inquiries into the role of *O*-GlcNAc cycling in stem cells have largely focused on altering *Oga* or *Ogt* in cultured embryonic stem cells. However, little is known about the function of *O*-GlcNAc cycling in an *in vivo* adult stem cell population. To test our hypothesis that *O*-GlcNAc cycling supports HSC function and immune homeostasis, we decided to specifically delete *Oga* in the hematopoietic lineage and assess the consequences on HSC maintenance and differentiation. Our laboratory previously generated a floxed allele of the *Oga* locus in mouse (*Oga*^*fl*/*fl*^)^16^. To make a hematopoietic lineage specific *Oga* mutant mouse, we bred the *Oga*^*fl*/*fl*^ with the Vav-iCre transgenic mouse^20^ from Jax (stock number 008610). The resulting mouse (*Oga*^*Vav*-*Cre*^) deleted *Oga* in HSCs. To ensure *Oga* deletion, we performed a fluorescence based OGA activity assay^21^ and found diminished OGA activity in bone marrow from *Oga*^*Vav*-*Cre*^ mice compared to their wildtype littermates (Fig. 1a). To test whether there was an increase in *O*-GlcNAc resulting from the *Oga* deletion, we assessed *O*-GlcNAc levels in bone marrow and liver by western blot. Here, we found elevated *O*-GlcNAc levels specifically in bone marrow from the *Oga*^*Vav*-*Cre*^ mice (Fig. 1b,c). Importantly, we were unable to detect *Oga* transcripts in Lin^−^Sca^+^Kit^+^ bone marrow cells from these mice (Supplementary Fig. S5), nor was there a change in endogenous *Vav* expression in these mutant cells (Supplementary Table S1). These experiments confirmed tissue-specific *Oga* deletion in HSCs from the *Oga*^*Vav*-*Cre*^ mice, resulting in increased *O*-GlcNAcylation.

**Figure 2:**
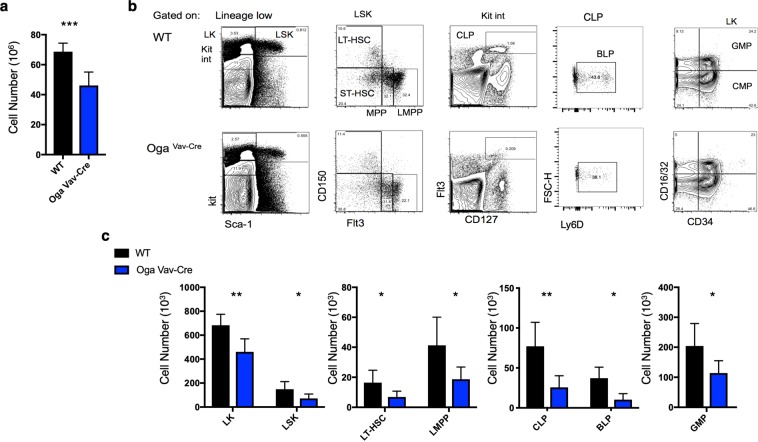
Reduced bone marrow progenitor populations in *Oga*^*Vav*-*Cre*^ mice. (**a**) Quantitation of total number of bone marrow cells from the indicated genotype. (**b**) Representative flow cytometric analysis, with boxes depicting gating, of long-term hematopoietic stem cells (LT-HSC, Lin^−^Sca1^+^Kit^+^ CD150^+^Flt3^−^), short-term hematopoietic stem cells (ST-HSC, Lin^−^Sca1^+^Kit^+^ CD150^−^Flt3^−^), multipotent progenitors (MPP, Lin^−^Sca1^+^Kit^+^ CD150^−^Flt3^+^) and lymphoid primed MPP (LMPP, Lin^−^Sca1^+^Kit^+^ CD150^−^Flt3^+^), common lymphoid progenitors (CLP, Lin^−^Kit^int^Flt3^+^CD127^+^), B cell-biased lymphoid progenitors (BLP, Lin^−^Kit^int^Flt3^+^CD127^+^Ly6d+) granulocyte/macrophage progenitors (GMP, Lin^−^Kit^+^Sca^−^CD16/32^+^CD34^+^) and common myeloid progenitors (CMP, Lin^−^Kit^+^Sca^−^CD16/32^−^CD34^+^). (**c**) Graphs depicting the absolute numbers of the indicated cell populations for the indicated genotype. Black bars represent wildtype and blue bars represent *Oga*^*Vav*-*Cre*^. N = 4–6, error bars represent standard deviation, *p < 0.05, **p < 0.01 as determined by t-test.

The hematopoietic system is comprised of increasingly specified progenitor cells, ultimately terminating in a fully differentiated lineage. Each of these distinct cell populations display unique cell surface markers that allow analysis by flow cytometry. In order to assess the consequences of *Oga* deletion on HSC maintenance and differentiation we analyzed HSC and progenitor cell populations in bone marrow from *Oga*^*Vav*-*Cre*^ mice in comparison to their wildtype littermates. The *Oga*^*Vav*-*Cre*^ mice had a significant decrease in total bone marrow cells as compared to wildtype (Fig. 2a). Using flow cytometry analysis, we detected significantly decreased progenitor and HSC populations, including the LK (Lin^−^Kit^+^) and the LSK (Lin^−^Sca-1^+^Kit^+^) progenitor populations (Fig. 2b,c). When gated on the LSK, further analysis indicated that the majority of the deficiency of the LSK population resulted from ~50% decrease in the long-term HSC (LT-HSC, CD150^+^Flt3^−^) and lymphoid-primed multipotent progenitor (LMPP, CD150^+^Flt3^+^) populations (Fig. 2b,c). Reduced LT-HSC pools suggested that deletion of *Oga* impaired maintenance of these stem cells. Investigation of further specified progenitor populations also uncovered significant reductions in common lymphoid progenitors (CLP, Kit^int^Flt3^+^CD127^+^) (Fig. 2b,c) and granulocyte-macrophage progenitors (GMP, Lin^−^Kit^+^CD16/32^+^CD34^+^) (Fig. 2b,c). Further analysis of the CLP population using Ly6D, a marker of B cell progenitors (BLP)^22^, indicated significant decreases in this population as compared to wildtype (Fig. 2b,c). Although total cell numbers were dramatically decreased in the mutant mice, the only population that was significantly decreased in relative frequency was CLP, indicating that the lymphoid lineage was particularly sensitive to *Oga* deletion (Supplementary Fig. S1a). Together, these data indicated that OGA was required for normal HSC maintenance and that without OGA there were substantial decreases in the HSC pools as well as further differentiated progenitor cell populations.

### Oga deficiency resulted in impaired thymocyte development

*O*-GlcNAc addition by the activity of OGT has an established role in T cell development and activation^15,18,23–27^. Early thymic progenitor cells are CD4 and CD8 double negative (DN), and T cell receptor (TCR) negative. DN thymocytes are further subdivided based on the expression of surface markers. DN1 cells are heterogeneous and can give rise to T cells, NK cells, B cells and macrophages. TCR rearrangement begins in DN2 cells. Commitment to the T cell lineage occurs in the transition from the DN2 to DN3 stage. DN3 cells that have successfully rearranged their TCRβ-chain, form a pre-TCR complex with a pre-TCRα chain^28^. This pre-TCR enforces β-selection and supports proliferation and differentiation, transitioning cells from DN3 to DN4. This process increases glucose and glutamine uptake with a concomitant increase in *O*-GlcNAc levels^18^. In fact, mice that conditionally deleted *Ogt* had a phenotype consistent with a failure of β-selection and had decreased numbers of thymocytes as well as decreased mature CD4+ and CD8+ T cells^15,18^.

**Figure 3:**
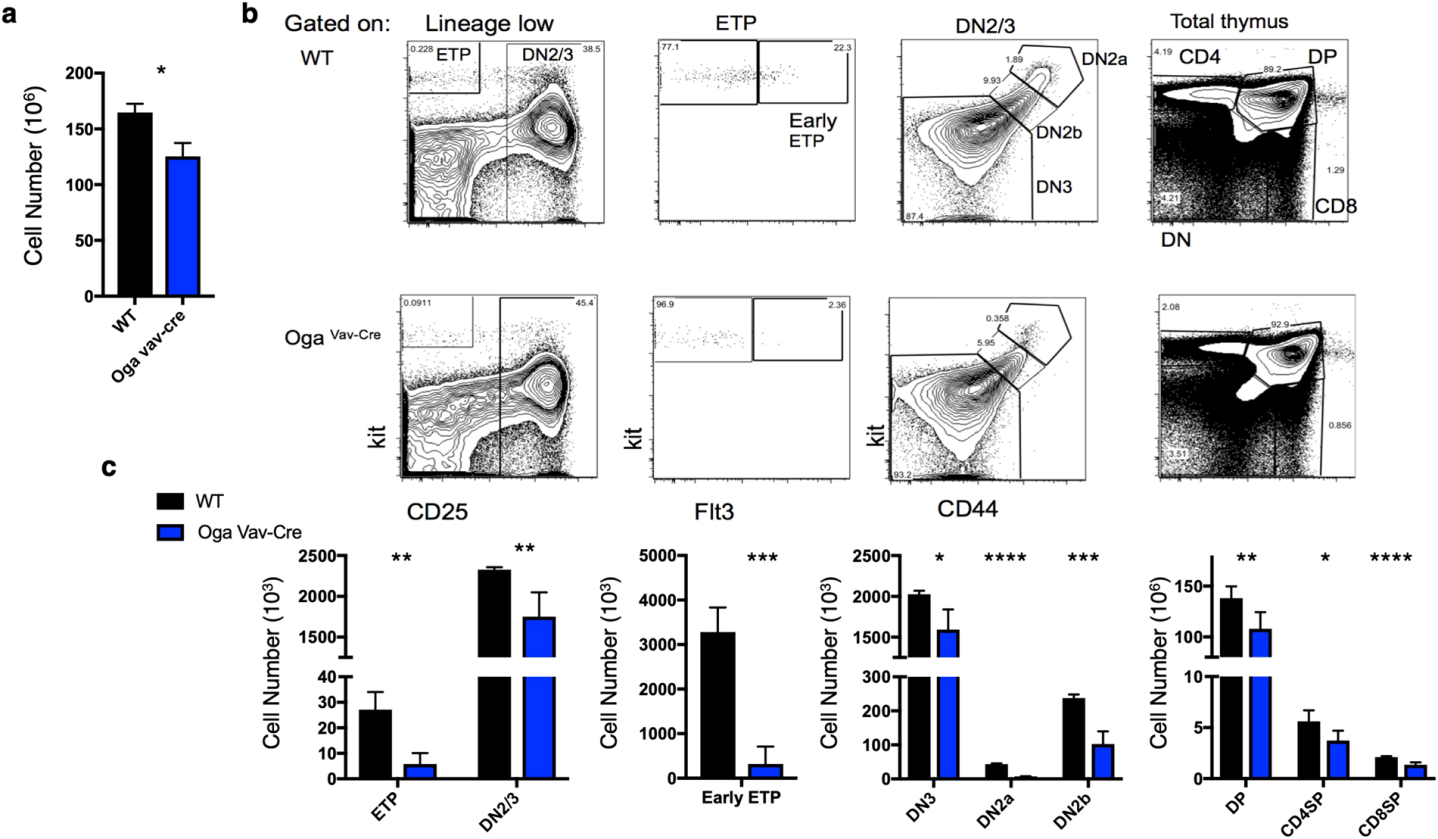
Decreased thymocytes in *Oga*^*Vav*-*Cre*^ mice. (**a**) Quantitation of total number of thymocytes from the indicated genotype. (**b**) Representative flow cytometric analysis, with boxes depicting gating of thymus cells including early thymic progenitors (ETP, Kit^+^CD25^−^) DN2/3 (CD25^+^), Early ETPs (Kit^+^CD25^−^Flt3^+^), DN2a (CD25^+^Kit^+^CD44^+^), DN2b (CD25^+^Kit^int^CD44^+^), DN3 (CD25^+^Kit^−^CD44^−^) and analysis of double positive (DP) CD8+ and CD4+ T cells. (**c**) Graphs depicting the absolute numbers of the indicated cell populations for the indicated genotype. Black bars represent wildtype and blue bars represent *Oga*^*Vav*-*Cre*^. N = 4–6, error bars represent standard deviation, *p < 0.05, **p < 0.01, ***P < 0.001, ****P < 0.0001 as determined by t-test.

**Figure 4:**
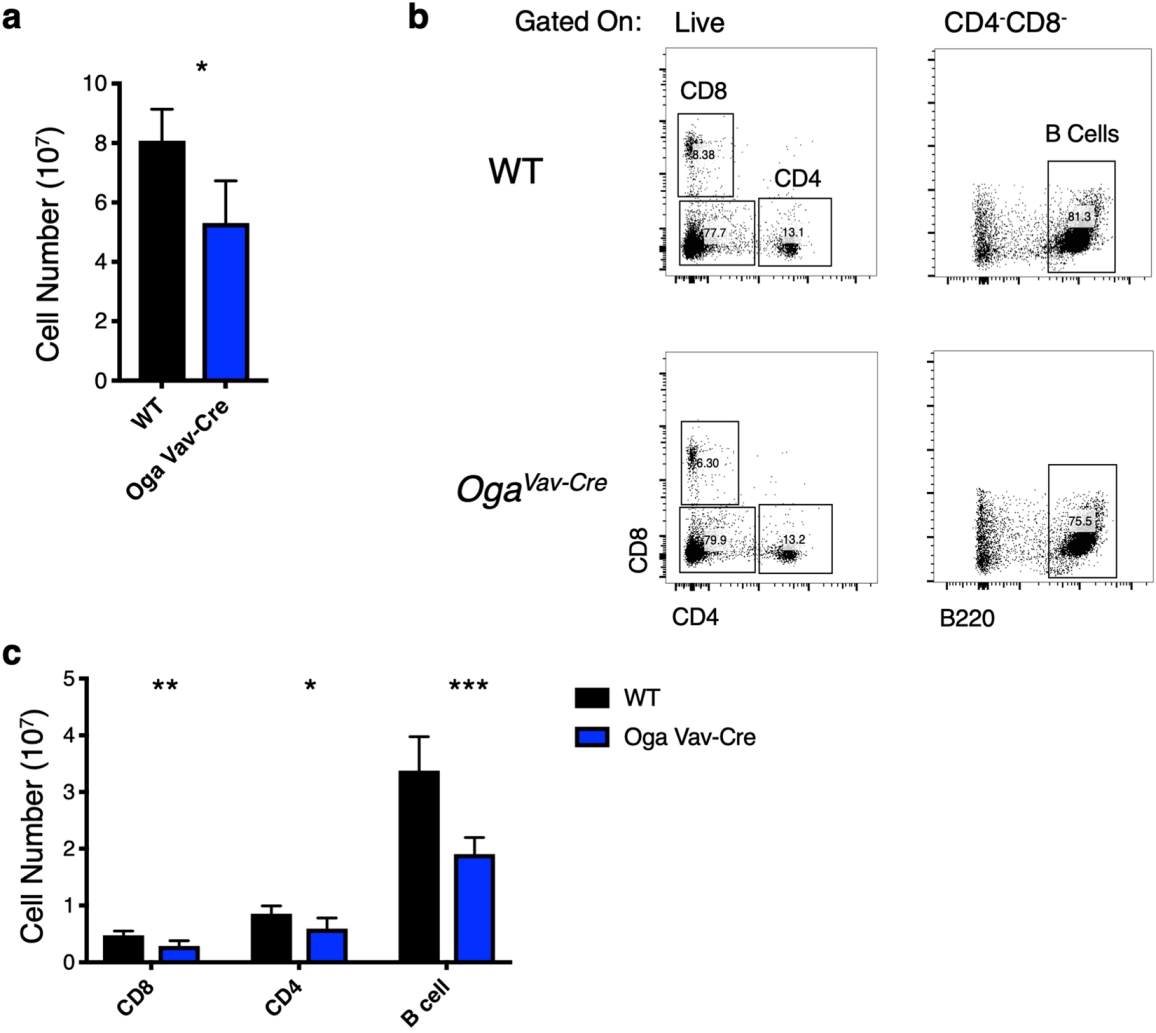
Reduced peripheral lymphoid populations in *Oga*^*Vav*-*Cre*^ mice. (**a**) Quantitation of total number of splenocytes from the indicated genotype. (**b**) Representative flow cytometric analysis of CD8^+^, CD4^+^ T cells, and B cell (CD4^−^CD8^−^B220^+^) populations from mouse spleen from the indicated genotype. (**c**) Graph depicting the absolute numbers of the indicated cell populations for the indicated genotype. Black bars represent wildtype and blue bars represent *Oga*^*Vav*-*Cre*^. N = 6, error bars represent standard deviation, *p < 0.05, **p < 0.01, ***P < 0.001, as determined by t-test.

While OGT had been previously shown to be important for T cell development, it has remained unknown how blocked *O*-GlcNAc cycling might impact this process. To answer this question, we systematically investigated thymocyte development in the *Oga*^*Vav*-*Cre*^ mice as compared to wildtype littermates. *Oga*^*Vav*-*Cre*^ mice had significantly decreased numbers of thymocytes (Fig. 3a). Consistent with the decrease in lymphoid progenitor populations, analysis of thymocyte populations by flow cytometry revealed a significant decrease in the cell number of all populations (Fig. 3b,c), including all DN populations, as well as the CD4/CD8 double positive (DP) and single positive populations. Most strikingly, there was also a significant decrease in frequency in the earliest detectable thymic progenitors (early ETP, Lin^−^Kit^+^CD25^−^Flt3^+^) as well as the DN2a and DN2b (Lin^−^Kit^+^CD25^+^CD44^+^) pools (Fig. 3b, Supplementary Fig. S1a). Further analysis of the spleen to assess peripheral populations indicated decreased splenocytes as well as decreased populations of CD8, CD4 T cells and B cells (Fig. 4a–c). These data suggest that the early HSC loss resulted in decreased cell populations that were maintained throughout development and in movement to the periphery.

### Impaired self-renewal and competitive repopulation capacity of Oga^Vav-Cre^ HSCs

**Figure 5:**
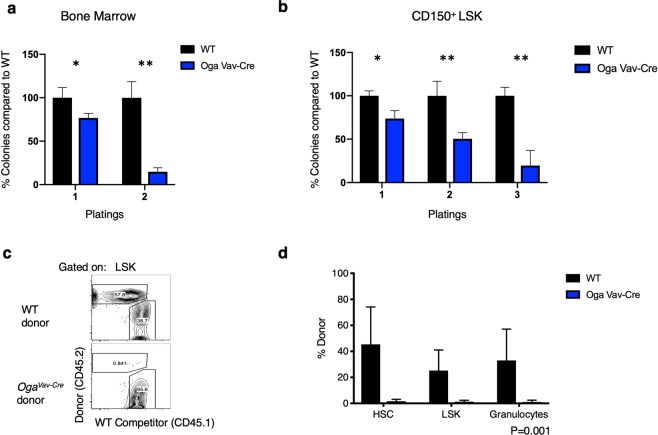
Impaired self-renewal and diminished competitive repopulation capacity of *Oga*^*Vav*-*Cre*^ HSCs. (**a**) 3 × 10^4^ bone marrow cells were plated in methylcellulose from the indicated genotype and colonies were counted 10–14 days after plating. For 2^nd^ plating, 1 × 10^4^ cells were plated with colonies counted at 10–14 days after re-plating. Number of wildtype colonies were set to 100% and number of mutant colonies were graphed accordingly. (**b**) Bone marrow cells from the indicated genotype were sorted to obtain LT-HSCs (Lin^−^Sca1^+^Kit^+^ CD150^+^Flt3^−^). 500 LT-HSCs were plated in methylcellulose and number of colonies were counted at 10–14 in culture. For re-plating, 1 × 10^4^ cells were re-plated and counted 10–14 days in culture. Number of wildtype colonies were set to 100% and number of mutant colonies were graphed accordingly. (**a**,**b**) Black bars represent wildtype and blue bars represent *Oga*^*Vav*-*Cre*^. N = 3, error bars represent standard deviation, *p < 0.05, **p < 0.01 as determined by student’s t-test. (**c**,**d**) CD45.1^+^ mice were irradiated and reconstituted with wildtype (WT) or *Oga*^*Vav*-*cre*^ bone marrow progenitors (CD45.2^+^) mixed with equal numbers of WT CD45.1^+^ bone marrow progenitors. CD45.1 and CD45.2 were used to distinguish between donor and WT competitor-derived cells from bone marrow after 10 weeks of reconstitution by flow cytometry. (**c**) Representative plot showing donor chimerism in the LSK compartment at 10 weeks of reconstitution. (**d**) Donor chimerism in HSC, LSK and one downstream cell lineage (granulocytes). (**c**,**d**) Black bars represent wildtype and blue bars represent *Oga*^*Vav*-*Cre*^. N = 3, error bars represent standard deviation, P = 0.001 as determined by 2-way ANOVA.

Two key characteristics of HSCs is the ability to self-renew and differentiate throughout the lifetime of an organism. Despite substantially reduced numbers of bone marrow HSCs, the surviving HSCs were able to differentiate (Figs 2–4). Accordingly, we observed normal *in*-*vitro* T cell differentiation from LSKs isolated from the *Oga*^*Vav*-*Cre*^ mice (Supplemental Fig. S2). Thus, we next wanted to directly test the impacts of OGA deletion on self-renewal and repopulation capacity. In order to examine the ability of *Oga* deficient HSCs to self-renew, we turned to an *in*-*vitro* colony forming unit (CFU) assay. For this experiment we plated whole bone marrow cells as well as sorted LT-HSCs (CD150^+^ LSK cells) isolated from *Oga*^*Vav*-*Cre*^ and wildtype mice. After 10–14 days of growth in methylcellulose, we found ~20% decrease in the number of colonies from the *Oga*^*Vav*-*Cre*^ derived cells versus the wildtype for both the whole bone marrow and LT-HSCs (Fig. 5a,b). To assess self-renewal, these cells were re-plated in methylcellulose and incubated again to form colonies. After re-plating, there was a greatly diminished ability of the *Oga*^*Vav*-*Cre*^ cells in the colony forming assay as compared to wildtype. Cells derived from whole bone marrow had ~80% decrease in number of colonies formed compared to wildtype and the LT-HSCs had ~50% decrease (Fig. 5a,b). After plating for a third time, the LT-HSCs had ~80% decrease in number of colonies formed as compared to wildtype (Fig. 5b). There were not enough *Oga*^*Vav*-*Cre*^ whole bone marrow cells to allow for a third plating. The decreases in colony numbers were not enriched for a specific cell-type (Supplementary Fig. S3). Therefore, these findings support the conclusion that blocked O-GlcNAc cycling impaired self-renewal ability of HSCs.

In order to directly assess competitive fitness of the *Oga*^*Vav*-*Cre*^ HSCs, we set up competitive bone marrow chimera mice. Here, we assessed the ability of *Oga*^*Vav*-*Cre*^ cells to engraft and reconstitute the hematopoietic system while in competition with wildtype cells. CD45.1^+^ mice were irradiated and reconstituted with wildtype or *Oga*^*Vav*-*Cre*^ bone marrow progenitor cells (CD45.2^+^) mixed with equal numbers of wildtype CD45.1^+^ bone marrow progenitor cells. CD45.1 and CD45.2 were used to distinguish between donor and wildtype competitor-derived cells by flow cytometry. We first analyzed blood granulocytes 5 weeks after reconstitution. We found that both wildtype as well as heterozygote donor cells were able to effectively compete with wildtype recipient cells, whereas the *Oga*^*Vav*-*Cre*^ did not contribute (Supplementary Fig. S4). Next, we did a more thorough analysis at 10 weeks after reconstitution and found that wildtype donor cells competed effectively with the wildtype recipient cells to reconstitute ~45%, 25% and 33% of the HSC, LSK, and granulocytes (Fig. 5c,d) of the chimeric mice, respectively. However, OGA depleted cells only reconstituted ~1% of the HSC, LSK, and granulocytes in the chimeric mice (Fig. 5c,d). These data suggested severe impairment of repopulation ability resulting from a decline in HSC maintenance in the mutants at both 5 and 10 weeks after the competitive bone marrow chimeras were initiated.

### Increased apoptosis in Oga^Vav-Cre^ bone marrow populations

**Figure 6:**
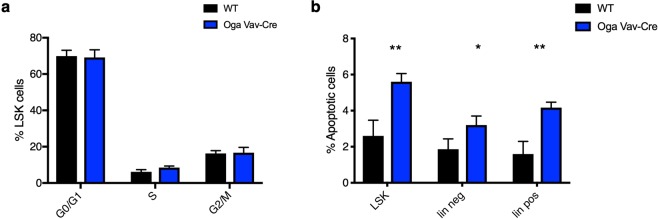
Normal proliferation but increased apoptosis of *Oga*^*Vav*-*Cre*^ bone marrow progenitors. (**a**) Percentage of LSK cells in the indicated cell cycle stage from each genotype was measured by DAPI staining and quantified by flow cytometry. Representative flow cytometric analysis can be found in Supplemental Fig. S3a. (**b**) Apoptosis was determined for the indicated genotypes and cell populations, by Annexin V positive and PI negative staining quantified by flow cytometry analysis. Representative flow cytometric analysis can be found in Supplemental Fig. S3b. Black bars represent wildtype and blue bars represent *Oga*^*Vav*-*Cre*^. N = 3, error bars represent standard deviation, *p < 0.05, **p < 0.01 as determined by t-test.

To better define the underlying causes of the HSC deficiency in the *Oga*^*Vav*-*Cre*^ mice, we assessed proliferation and apoptosis of the mutant LSKs versus wildtype. Both a decrease in proliferation and an increase in proliferation can lead to a loss of HSC populations and diminished competitive repopulation capacity. Whereas a decrease in proliferation gives the wildtype competitors a distinct advantage, a highly proliferative HSC pool causes HSC exhaustion because these cells should be largely quiescent^29^. Alternatively, an increase in apoptosis would also deplete cell populations, impair self-renewal and give a competitive advantage to wildtype cells. To assess these possibilities, we performed cell cycle analysis by DAPI staining on LSK cells isolated from wildtype or *Oga*^*Vav*-*Cre*^ mice and both had similar cell cycle profiles (Fig. 6a, Supplemental Fig. S5a).

Next, we analyzed apoptosis in the LSK populations by Annexin V and PI staining. The *Oga*^*Vav*-*Cre*^ cells showed approximately double the number of apoptotic LSK cells as their wildtype counterparts (Fig. 6b, Supplemental Fig. S5b), with an increase in apoptosis from about 2.5% to 5%. To assess if this effect on apoptosis was specific to the stem cell population we also analyzed Annexin V and PI staining in all lineage negative populations as well as the lineage positive population, and found a similar doubling of apoptotic cells, from about 2% to 4%. This increase in apoptosis without any change in proliferation rate likely contributed to the competitive disadvantage of the mutant cells as well as the decrease in cell populations.

### Transcriptional deregulation of genes in Oga^Vav-Cre^ progenitor cells

**Figure 7:**
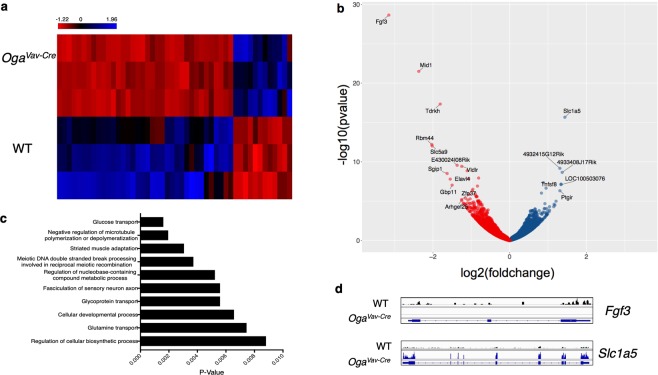
Transcriptional deregulation of genes involved in stem cell function. RNA-Seq was performed on sorted LSK cells from 3 *Oga*^*Vav*-*Cre*^ and 3 wildtype mice. Genomatix analysis called 41 significantly downregulated and 12 significantly upregulated genes represented as a heatmap (using RPKMs) (**a**) and volcano plot (**b**). (**b**) *Oga* was excluded from volcano plot for scale, genes with a log2(foldchange) ± >1.25 and p < 0.005 are labelled. (**c**) Top 10 enriched gene ontology terms (GO) among the deregulated genes as determined by Genomatix. (**d**) Representative IGV view of the most significantly down regulated (*Fgf3*) and upregulated (*Slc1a5*) genes.

Our data indicated that hyper-*O*-GlcNAcylation resulted in increased apoptosis, HSC dysfunction and a decline in stem cell maintenance. Both stem cell maintenance and differentiation require tight transcriptional regulation. In fact, *O*-GlcNAc is essential for proper transcriptional control by directly modifying RNA POLII^30–32^, modifying histones^33–36^ and through interactions with epigenetic regulators like PcG^8–11^, TRX^8,12,13^, and TET^37–40^ proteins. Interestingly, microarray analysis of *Oga*^−/−^ mouse embryonic fibroblasts (MEFs) indicated transcriptional deregulation of genes necessary for HSC maintenance including Hif1α, Tal1, Lyl1 and Cdkn1c^16^. Therefore, we hypothesized that in addition to the increased apoptosis, transcriptional deregulation also contributed to HSC dysfunction of *Oga* deficient HSCs. To assess whether there were transcriptional changes in the *Oga* mutants, we performed RNA-Seq on sorted LSK cells derived from the bone marrow of *Oga*^*Vav*-*Cre*^ mice in comparison to wildtype. We identified 41 significantly downregulated and 12 upregulated genes (Fig. 7a,b, Supplemental Table S1). Bioinformatic analysis using Genomatix indicated enrichment of pathways involved in HSC function within the deregulated genes. Interestingly, 3 out of the 10 most significantly deregulated gene ontology (GO) terms (Fig. 7c) had to do with nutrient uptake and signaling (glucose transport, glycoprotein transport and glutamine transport). HSCs rely significantly on glycolysis and glutaminolysis for their energy needs and changes in glucose and glutamine uptake could have consequences on metabolic regulation. Further, the most significantly upregulated (*Slc1a5*) and downregulated (*Fgf3*) genes (Fig. 7b,d, Supplemental Fig. S6) have defined roles in self-renewal and lineage specification of adult stem cells^41,42^. Thus, these data suggested that *O*-GlcNAc cycling contributes to transcriptional networks regulating signaling and nutrient uptake in hematopoietic stem cells.

## Discussion

Stem cells are characterized by their unique metabolic profiles and transcriptional networks. Their reliance on glycolysis allows for the production of metabolic intermediates that provide the building blocks for growth, and contributes to survival in a hypoxic environment. Tight transcriptional regulation enforces the pluripotency state of stem cells and permits controlled fate specification. At the nexus of these processes is *O*-GlcNAcylation, which integrates metabolism with cell physiology. Physiologically, the regulation of *O*-GlcNAc cycling is quite complex. Levels of *O*-GlcNAc are regulated in a number of ways including; (1) feedback inhibition of UDP-GlcNAc levels^43^, (2) microRNAs^44–46^, (3) a unique intron mechanism which limits the levels of OGT produced^47^ and (4) varying levels and activities of OGA. This last mechanism involving the regulated expression of *O*-GlcNAcase is certain to play a pivotal role in regulating the physiological levels of *O*-GlcNAc. Here, we focused on deleting *Oga* to assess the impact of *O*-GlcNAc cycling on HSC homeostasis and early T cell development.

Our lab, and others, have shown that OGA is essential for normal differentiation of mouse embryonic stem cells^6,7^. The *Oga* gene is located in the highly evolutionarily conserved *Lbx1*/*Ladybird* NK cluster. The *Lbx* genes have essential roles in development, including key functions in neural and mesodermal specification^48^. Despite having a clear role in embryonic stem cell biology, it’s *in vivo* role in adult stem cells had not previously been investigated. Because knocking out *Ogt* or *Oga* in the mouse is lethal^5,15,16^, we analyzed a tissue-specific deletion of *Oga*. This was done by crossing the *Oga*^fl/fl^ mice with Vav-iCre mice to conditionally delete *Oga* in HSCs. Thus, while OGT could properly modify proteins, this modification could not be removed in the hematopoietic lineage of these mutant mice. HSCs differentiate towards increasingly specified progenitors, with distinct cell surface markers that allowed for quantitation by flow cytometry. We found that *Oga*^*Vav*-*Cre*^ mice had a substantially decreased HSC population as well as diminished numbers of intermediate progenitors. *In*-*vitro* CFU assays  indicated that the *Oga* deficient HSCs had impaired self-renewal compared to wildtype. Further, when put in competition with wildtype HSCs, the mutant HSCs were unable to contribute to repopulation of an irradiated mouse. Together these data suggested that OGA was required for normal HSC function and that elevated *O*-GlcNAc had consequences on HSC maintenance, fitness and competitive repopulation capacity.

*O*-GlcNAc is required in stem cells to maintain the pluripotency transcriptional network, and has been proposed to be an essential component regulating stem cell self-renewal by modifying transcription factors like OCT4, cMYC and SOX2^6^. Previous transcriptional analysis of *Oga*^−/−^ MEFs found significant enrichment of deregulated genes associated with immunity and metabolism^16^ suggesting a possible regulatory role for *O*-GlcNAc cycling in the hematopoietic lineage. In fact, a recent report suggested a link between transcriptional regulation via *O*-GlcNAc cycling with *in vitro* erythropoiesis^17^. To further define a possible mechanism for HSC dysfunction, we performed RNA-Seq and qRT-PCR confirmation to uncover deregulated gene expression in LSK cells. Similar to the MEF transcriptional data, we also found a significant enrichment of genes involved in development and nutrient signaling as deregulated. Importantly, the most significantly down regulated and up regulated genes in the OGA depleted LSK cells have defined roles in adult stem cell maintenance and lineage specification. One such gene was *Fgf3*, a canonic member of the fibroblast growth factor family. FGF signaling plays critical roles in adult stem cell self-renewal^41^ and specifying primitive hematopoietic stem cells in the embryo^49,50^. While most FGFs are secreted, FGF3 is both secreted and transported to the nucleus where it plays a role in controlling cell growth^51–53^.

The metabolic state, including glucose and glutamine metabolism, of HSCs is important for both self-renewal and lineage specification and commitment. Our RNA-Seq analysis found deregulation of factors involved in both glucose and glutamine transport. The most highly upregulated gene, *Slc1a5*, is a glutamine transporter required in HSCs for erythyroid specification^42^. Further, two genes involved in glucose consumption, *Clip3*^54^ and *Slc5a9*^55^ were significantly downregulated in the *Oga* mutant LSK cells. HSCs require a distinct metabolic profile for self-renewal and differentiation. Our study suggests that interference with *O*-GlcNAc cycling disturbed the transcriptional network regulating nutrient uptake in the LSK cells.

Interestingly, glutamine enters the HBP and increased glutamine uptake could increase *O*-GlcNAcylation. Thus, upregulation of *Slc1a5* expression in the LSK cells suggests a positive feedback loop whereby increased *O*-GlcNAcylation results in upregulation of glutamine transporters and possibly increasing flux through the HBP. Other studies have also described an *O*-GlcNAc dependent positive feedback loop for metabolites entering the HBP. For example, *O*-GlcNAcylation of cMYC in T cells increased *Glut1* expression in mouse T cells. With increased *Glut1* there was increased glucose uptake, ultimately contributing towards *O*-GlcNAcylation of cMYC^18^.

Aside from transcription factors and epigenetic regulators known to be *O*-GlcNAc modified that are involved in HSC regulation, analysis of about 150 mouse mutants with HSC phenotypes has highlighted the role of specific pathways involved in HSC homeostasis^29^. Interestingly, *O*-GlcNAc cycling is intimately involved in these pathways which include cell cycle^56^, TGF-β^57,58^, PTEN/AKT^59^, and WNT^60,61^ signaling pathways. Thus it was likely that blocked *O*-GlcNAc cycling influenced these pathways contributing to increased apoptosis and disruption of HSC homeostasis. Unfortunately, due to limited material of these very early cell populations, further biochemical characterization was difficult.

Analysis of the thymus revealed that early thymocytes were particularly sensitive to OGA deletion. The ETP and DN2 populations were the only populations analyzed that also had significant decreases in frequency in the mutant mice. This suggested that although the primary defect in these mice was in HSC maintenance, there was a secondary defect in the earliest stages of thymocyte development. Dynamic changes in *O*-GlcNAc had been demonstrated in early thymocyte development, with OGT being identified as a critical factor in these cells^15,18^. Here, we have identified removal of *O*-GlcNAc as an important component of early thymic development. Thus, we conclude that both the addition and the cycling of *O*-GlcNAc support normal T cell development.

Our data highlights the role dynamic *O*-GlcNAc cycling plays in HSC maintenance. We note that both the inability to compete in a bone marrow chimera assay and the greatly diminished numbers of early ETPs might suggest impaired homing could have contributed to the mutant phenotypes. Proper homing requires an exquisite interplay between chemokines, chemokine receptors, intracellular signaling, adhesion molecules and proteases. In fact, key cell adhesion molecules involved in homing, vascular cell adhesion molecule 1 (VCAM-1) and intracellular adhesion molecule (ICAM-1), have been previously reported to be indirectly regulated by *O*-GlcNAc through modification of transcription factors like NFκB^62^, SP1^63^ and TNFα^64^. In-depth analysis of our RNA-seq data from mutant LSKs revealed a small but significant increase in basal cell adhesion molecule (BCAM) (Supplemental Table 1). However, BCAM does not have a known role in homing, and bioinformatic analysis did not reveal cell adhesion as an enriched pathway. Further, as described above, altering *O*-GlcNAc cycling and transcription of nutrient transporters could alter flux through the HBP which has the potential to impact extracellular N-linked glycosylation. In fact, glycosylation of cell surface molecules is required for normal homing^65,66^. Thus, the interplay between intracellular and extracellular glycosylation, while quite interesting, will require further research.

Together, these experiments suggested that the processes of *O*-GlcNAc addition and removal each play a key role in regulating hematopoiesis. Loss of *Oga* in mouse HSCs resulted in an abnormal stem cell population with numbers of hematopoietic progenitors and further differentiated T and B cells remaining low. Furthermore, *Oga* deficient HSCs had impaired self-renewal, decreased repopulation capacity and an altered transcriptional profile. This is the first report to uncover *O*-GlcNAc cycling as a critical component of adult stem cell maintenance *in vivo* and has important implications for the role of hexosamine signaling in the maintenance of adult stem cell populations giving rise to immune homeostasis.

## Methods

### Mice

The Vav-iCre transgenic mouse^20^ was purchased from Jax (stock number 008610). The hematopoietic specific *Oga* deletion was obtained by breeding the *Oga*^*fl*/*fl*^ described previously^16^ with the Vav-iCre. Genotyping for the floxed allele was confirmed using primer Oga 7F and Oga7R. Presence of Cre was confirmed using primers oIMR9266 and oIMR9267 (see Supplemental Table 2 for primer sequences). All mice were analyzed between 4–12 weeks of age. Both males and females were used in analysis. The animals were maintained according to the animal protocol # K023-LCBB-16 approved by the NIDDK Animal Care and Use Committee, National Institutes of Health.

### Oga assay

OGA assay was performed as described previously^21^ using 30ug of lysates. To control for any background fluorescence of the FDGlcNAc substrate itself, a no lysate control was subtracted from all samples. Fluorescence was measured in 1-s intervals at the excitation wavelength of 485 nm and emission wavelength of 520 nm on a Polarstar Omega (BMG Labtech). All assays were performed in triplicate.

### Western blot

Protein was extracted with T-PER tissue protein extraction reagent (Thermo-Fisher). Lysates were run on a 4–12% Bis-Tris gel and transferred to a nitrocellulose membrane. Membranes were blocked with Odyssey blocking buffer (Odyssey, number 927–40000) for 1 h, incubated with primary antibodies rabbit anti-actin (Abcam) and mouse anti-*O*-GlcNAc (RL2) (Thermo Fisher MA1-072) overnight at 4 °C. Secondary antibodies were Odyssey IR dye-conjugated. Membranes were imaged and band intensities were analyzed with Odyssey imaging equipment.

### Antibodies and flow cytometry

Cell suspensions were counted after ACK lysis using a Nexcelom Cellometer and incubated with a mix of purified rat, mouse and hamster IgG before addition of specific antibodies. Antibodies specific for B220 (RA3-6B3), CD19 (1D3), Mac-1 (M1/70), Gr-1 (8C5), CD11c (N418), Ter119 (TER119), NK1.1 (PK136), CD3ε (2C11), CD4 (GK1.5), CD8α (53-6.72), CD8β (H35-17.2), TCRβ (H57), TCRγδ (GL-3), Kit (2B8), Sca-1 (D7), CD150 (mShad150), CD34 (RAM34), CD16/32 (93), IL-7Rα (A7R34), CD25 (PC61.5), CD44 (IM7), Thy-1.2 (53-2.1), Ly6d were from Thermo Fisher, and anti-Flt3 (A2F10) was from BD. The lineage ‘cocktail’ (Lin) is a mix of the following antibodies: anti- B220, CD19, Mac-1, Gr-1, CD11c, Ter119, NK1.1, CD3ε, CD8α, CD8β, TCRβ and TCRγδ. Live/dead discrimination was performed by staining with DAPI. Annexin V and PI were from Thermo Fisher. Samples were acquired using an LSRFortessa flow cytometer (BD) and analyzed using FlowJo software (Tree Star). All analyses are presented on singlet live cells. Total cell numbers were determined by multiplying total cell count by the frequency of the specific population.

### *In*-*vitro* colony forming assays

Bone marrow was isolated from *Oga*^*Vav*-*Cre*^ and wildtype littermates and after ACK lysis were seeded at a density of 3 × 10^4^ cells/replicate or sorted to obtain CD150^+^LSK cells and seeded at a density of 500 cells/replicate in methocult M3434 (Stemcell Technologies). Colonies propogated in culture were scored at 10–14 days. Cells were resuspended and 1 × 10^4^/replicate were replated.

### Competitive chimera

Bone marrow cells were isolated from OGA deficient mice and littermate controls, lineage-depleted, and injected into lethally irradiated (850 rads) CD45.1 recipient mice with competitor CD45.1 bone marrow cells.

### RNA-Seq

Bone marrow cells collected from 3 *Oga*^*Vav*-*Cre*^ and 3 wildtype female mice were sorted for Lin^−^Sca^+^Kit^+^ cells using a BD FACSAria Fusion Flow cytometer and the following antibodies: Lineage Cocktail FITC (R&D Biosystems), Sca-1(D7)APC (ebiosciences), c-Kit (2B8)PE (ebiosciences), FVD (ebiosciences). At least 2 × 10^4^ cells were collected for each sample. RNA was extracted using RNeasy plus micro kit (Qiagen) following manufacturers protocol. RNA quality was confirmed using Bioanalyzer (Agilent technologies) all RIN scores were >7. RNA libraries were prepped using NEBNext Ultra RNA Library Prep Kit for Illumina and the NEBNext Poly(A) mRNA Magnetic Isolation Module (New England Biolabs). Single-end 50 bp reads were generated by a HiSeq2500 sequencer. Raw reads were aligned to the mouse genome (mm9) and bioinformatic analysis was done using Genomatix. Significance was determined by DESeq2 parametric Wald test^67^. Significant transcripts had an adjusted p-value < 0.05 and log2(fold-change) > 1 or <−1. Visualization was done using Partek Flow (Partek), R (R Core Team) and IGV^68^. RNA-Seq data are available at GEO under accession number GSE116620.

### qRT-PCR

cDNA libraries prepared for RNA-Seq were diluted 1:5. Fast SYBR Green Master Mix (Applied Biosystems) was used for amplification following manufacturers protocol using 2 ul of cDNA and the appropriate primer (Supplemental Table 2) on an 7900HT fast real-time PCR system (Applied Biosystems). Each reaction was performed in technical quadruplicate. Relative gene expression was normalized to the geometric mean of *Rplp0*, *Eef2*, *Rpl38* (Supplemental Table 2). Significance was determined using an unpaired t test. P < 0.05.

### *In vitro* T cell differentiation

OP9, OP9Dl1 and LSKs were cultured in α-MEM media supplemented with 20% FBS, glutamine, penicillin and streptomycin. For T cell–differentiation assays, LSK were cultured on irradiated OP9-Dl1 stromal layers in the presence of IL-7 (1 ng/ml) and Flt3L (5 ng/ml). All cytokines were purchased from PeproTech. Cultures were analyzed 15 days later for the presence of CD45.2^+^ hematopoietic progeny. Thy1-2^+^ CD25^+^ cells were considered as T lineage cells.

### Statistical analysis

Graphpad prism was used for all statistics. A two-tailed paired t test or a two way anova was used to determine significance as indicated in figure legends. P values less than .05 was considered statistically significant.

## Supplementary information

Supplementary Information

## Data Availability

RNA-Seq data are available at GEO under accession number GSE116620.
