# Supplementary material for: Blocked O-GlcNAc cycling disrupts mouse hematopoeitic stem cell maintenance and early T cell development
Source: Sci Rep. 2019 Aug 29;9:12569. doi: 10.1038/s41598-019-48991-8 (PMC6715813; doi:10.1038/s41598-019-48991-8)
Supplement: Supplementary file 1 — Supplementary Information [file 41598_2019_48991_MOESM1_ESM.pdf]

Blocked *O*-GlcNAc cycling disrupts mouse hematopoeitic stem cell maintenance and early T cell development

Lara K. Abramowitz[1], Christelle Harly[2,3], Arundhoti Das[2] Avinash Bhandoola[2], and John A. Hanover[1*]

[1]Laboratory of Cellular and Molecular Biology, National Institute of Diabetes and Digestive and Kidney Diseases, National Institute of Health, Bethesda, MD 20892, USA.

[2]Laboratory of Genome Integrity, Center for Cancer Research, National Cancer Institute, National Institutes of Health, Bethesda, MD 20892, USA.

[3]CRCINA, INSERM, CNRS, Université d'Angers, Université de Nantes, Nantes, France.

[*] Correspondance: John A. Hanover, Laboratory of Cellular and Molecular Biology, National Institute of Diabetes and Digestive and Kidney Diseases, National Institute of Health, Bethesda, MD 20892; e-mail: jah@helix.nih.gov;

Supplemental Figure Legends:

Supplemental Figure S1. Reduced frequency of lymphoid progenitors and early thymocytes from *Oga*$^{Vav\text{-}Cre}$ mice. (a) Graphs quantifying the percent of total cells of each corresponding cell population from the indicated genotype from the flow cytometry analysis of bone marrow. (b) Graphs quantifying the percent of total cells of each corresponding population from the indicated genotype from the flow cytometry analysis of the thymus. (c) Graphs quantifying the percent of total cells of each corresponding population from the indicated genotype from the flow cytometry analysis of the spleen. Black bars represent wildtype and blue bars represent *Oga*$^{Vav\text{-}Cre}$*.* N=4-6, error bars represent standard deviation, **p<.01, ***P<.001, as determined by t-test.

Supplemental Figure S2. Normal *in vitro* T cell differentiation of *Oga* mutant cells. Equal numbers of LSK cells were cultured for 15 days on OP9Dl1 to induce T cell differentiation. (a) Representative flow cytometry analysis. (b) quantitation of T cell numbers. Black bars represent wildtype and blue bars represent *Oga*$^{Vav\text{-}Cre}$*.* N=6, error bars represent standard deviation.

Supplemental Figure S3. Similar proportion of CFU types in CFU-forming assay. Bone marrow was isolated from wildtype (WT) or *Oga*$^{Vav\text{-}cre}$ mice, red blood cells were lysed and $3x10^4$ cells were plated in methylacellulose. Colonies were scored after incubating for 10-14 days. N=3, error bars represent standard deviation.

Supplemental Figure S4. Diminished competitive repopulation capacity of *Oga*$^{Vav\text{-}Cre}$ HSCs. $CD45.1^+$ mice were irradiated and reconstituted with wildtype (WT), *Oga*$^{fl/+;\ Vav\text{-}Cre}$ (Het), or *Oga*$^{Vav\text{-}cre}$ bone marrow progenitors ($CD45.2^+$) mixed with equal numbers of WT $CD45.1^+$ bone marrow progenitors. CD45.1 and CD45.2 were used to distinguish between donor and WT competitor-derived cells in blood after 5 weeks of reconstitution by flow cytometry. (a)

Representative plot showing donor chimerism in blood granulocytes 5 weeks after reconstitution. (b) quantitation of donor chimerism in blood granulocytes. Black bars represent wildtype, purple bars represent hets, blue bars represent *Oga*$^{Vav\text{-}Cre}$*.* N=3, error bars represent standard deviation.

Supplemental Figure S5. Increased apoptosis of *Oga*$^{Vav\text{-}Cre}$ bone marrow. Representative flow cytometry analysis to analyze cell cycle (a) and apoptosis (b) gated on LSK cells.

Supplemental Figure S6. Deregulation of genes in *Oga*$^{Vav\text{-}Cre}$ LSK cells. (a) Representative IGV view of *Oga* to confirm loss of *Oga* transcripts in the mutant mice. (b-c) qRT-PCR confirmation of (b) increased *Slc1a5* and (c) decreased *Fgf3* expression in *Oga*$^{Vav\text{-}Cre}$ LSK cells. Expression was normalized using the geometric mean of *Aarbpo*, *Eef2* and *Rpl38.* Black bars represent wildtype and blue bars represent *Oga*$^{Vav\text{-}Cre}$*.* N=3, error bars represent standard deviation, *$p<0.05$ as determined by t test.

Supplemental Figure S7. Full length blots from figure 2. *O*-GlcNAc was assessed by Western blot using lysates from liver and bone marrow of WT and *Oga*$^{Vav\text{-}Cre}$ (Vav-Cre) mice using RL2 for *O*-GlcNAc and β-Actin as loading control. Full length blots that were cut at 55kDa to allow for simultaneous quantitation of *O*-GlcNAc and β-Actin. Lanes used in figure 2 are indicated with the appropriate genotype and tissues the lysates were derived from.

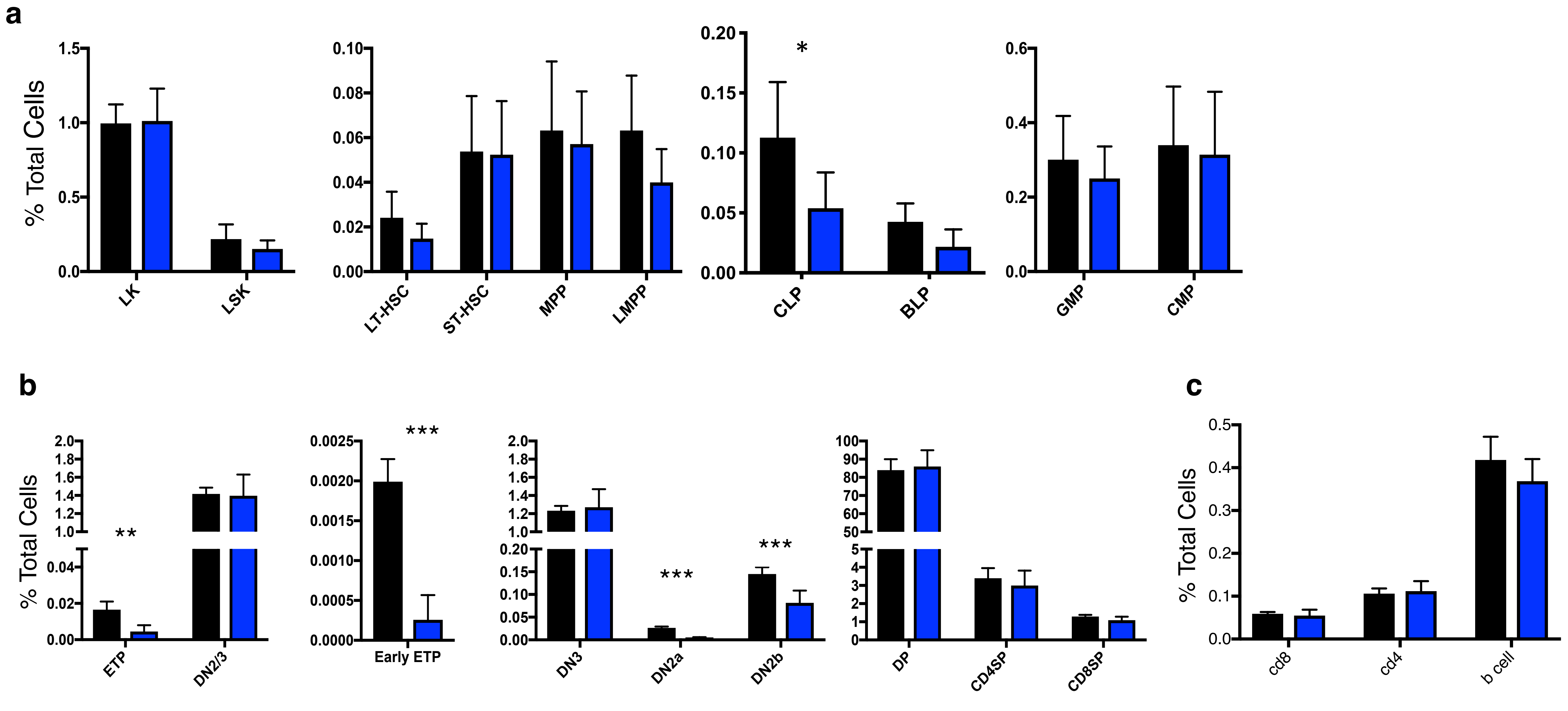

Supplemental Figure S1

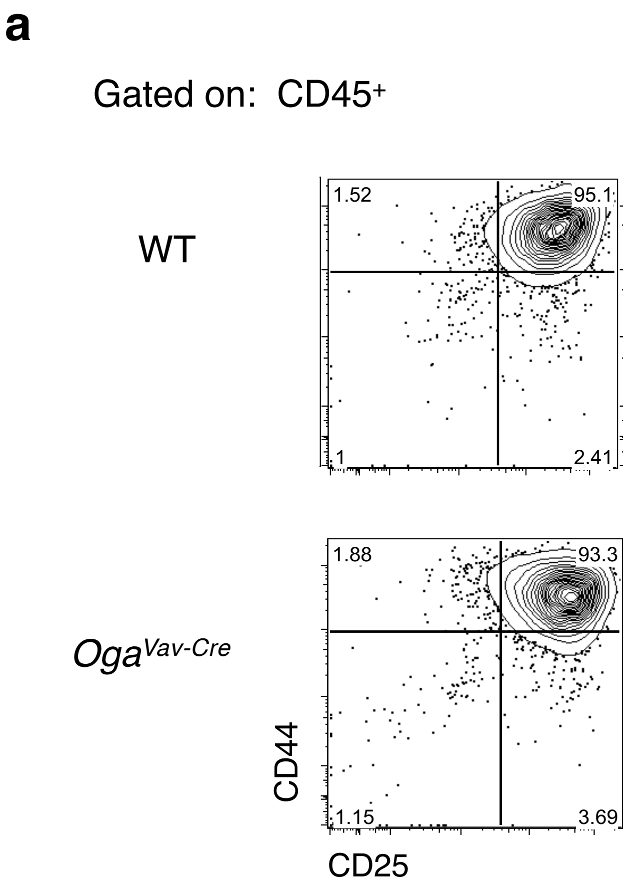

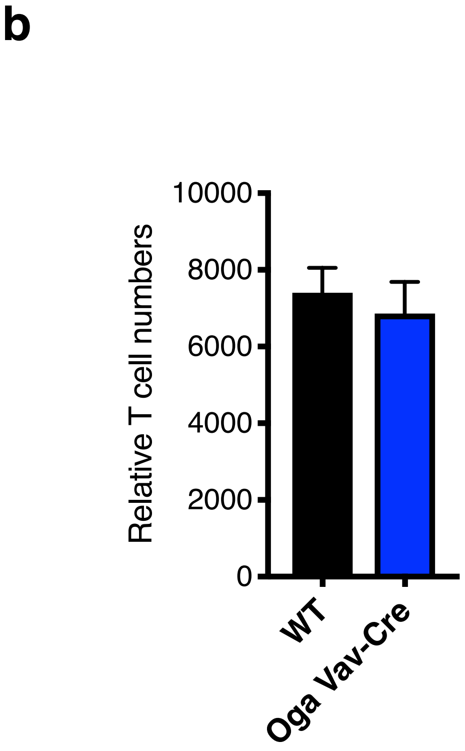

Supplemental Figure S2

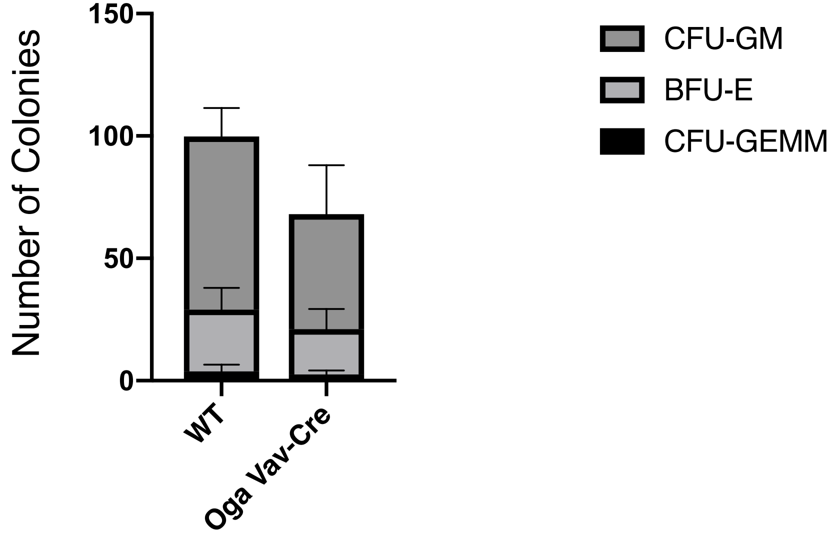

Supplemental Figure S3

a

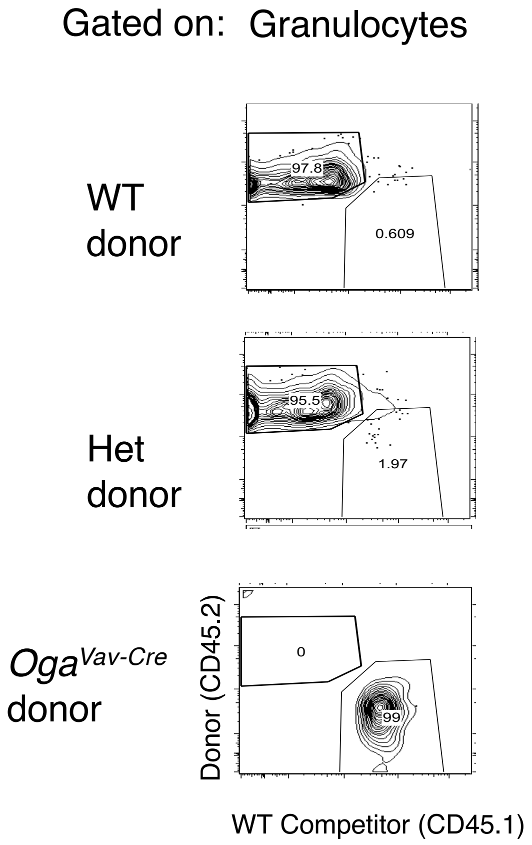

b

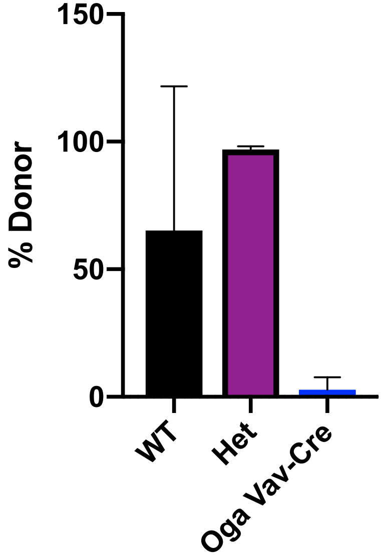

Supplemental Figure S4

a

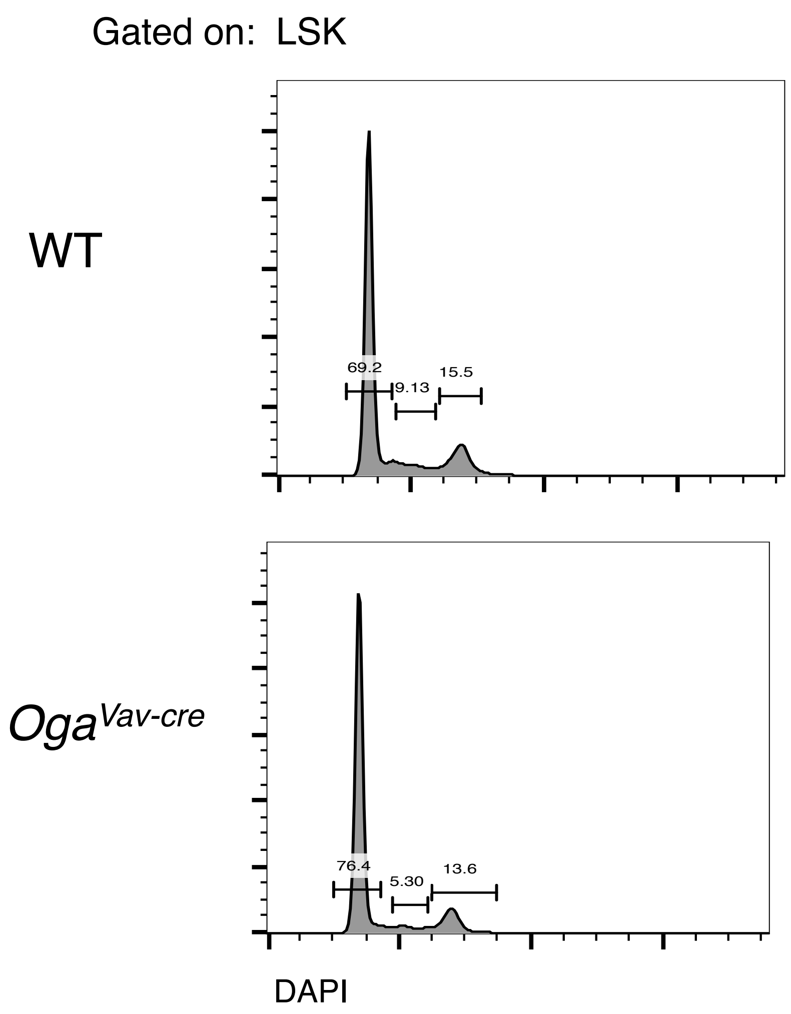

b

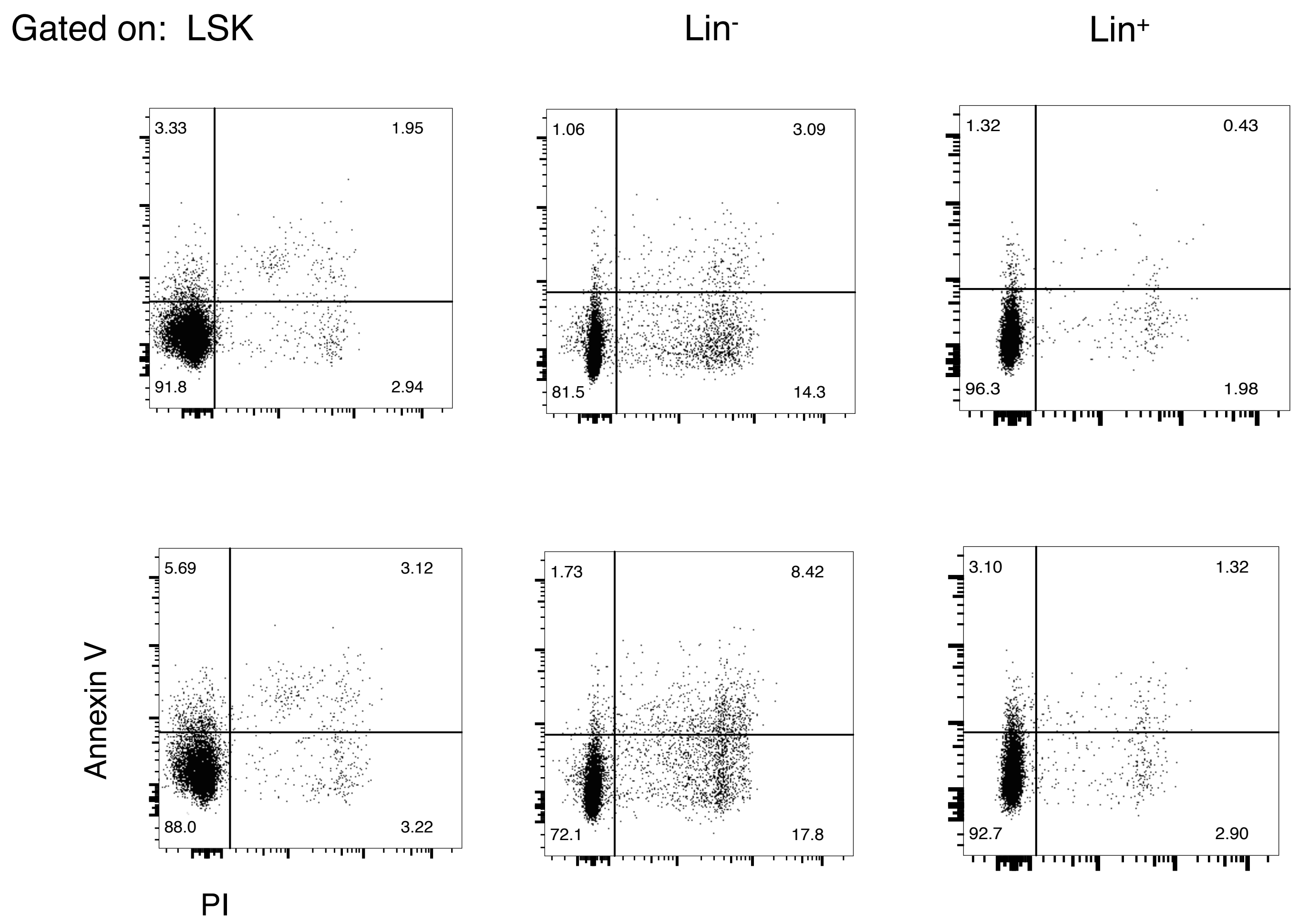

Supplemental Figure S5

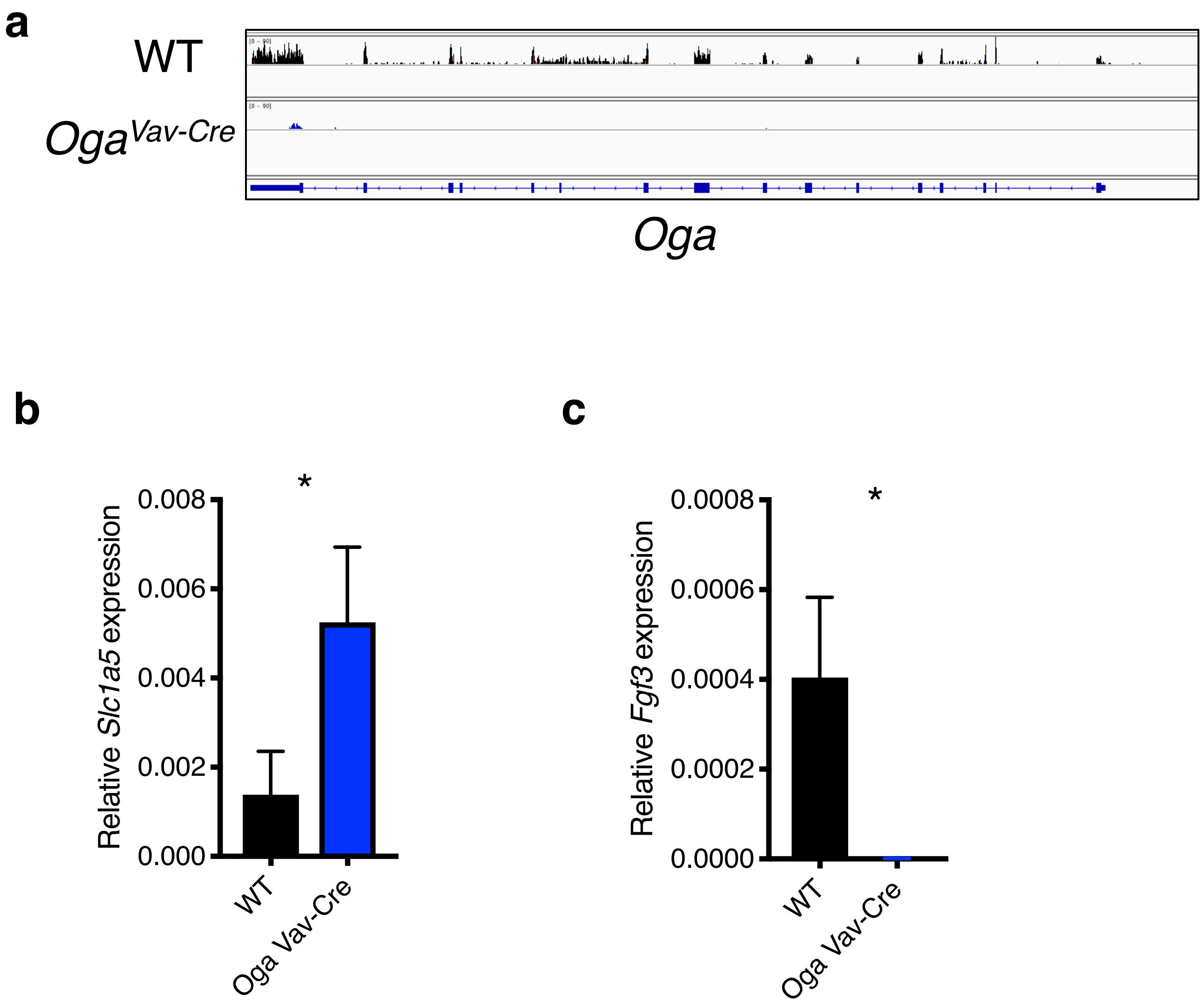

Supplemental Figure S6

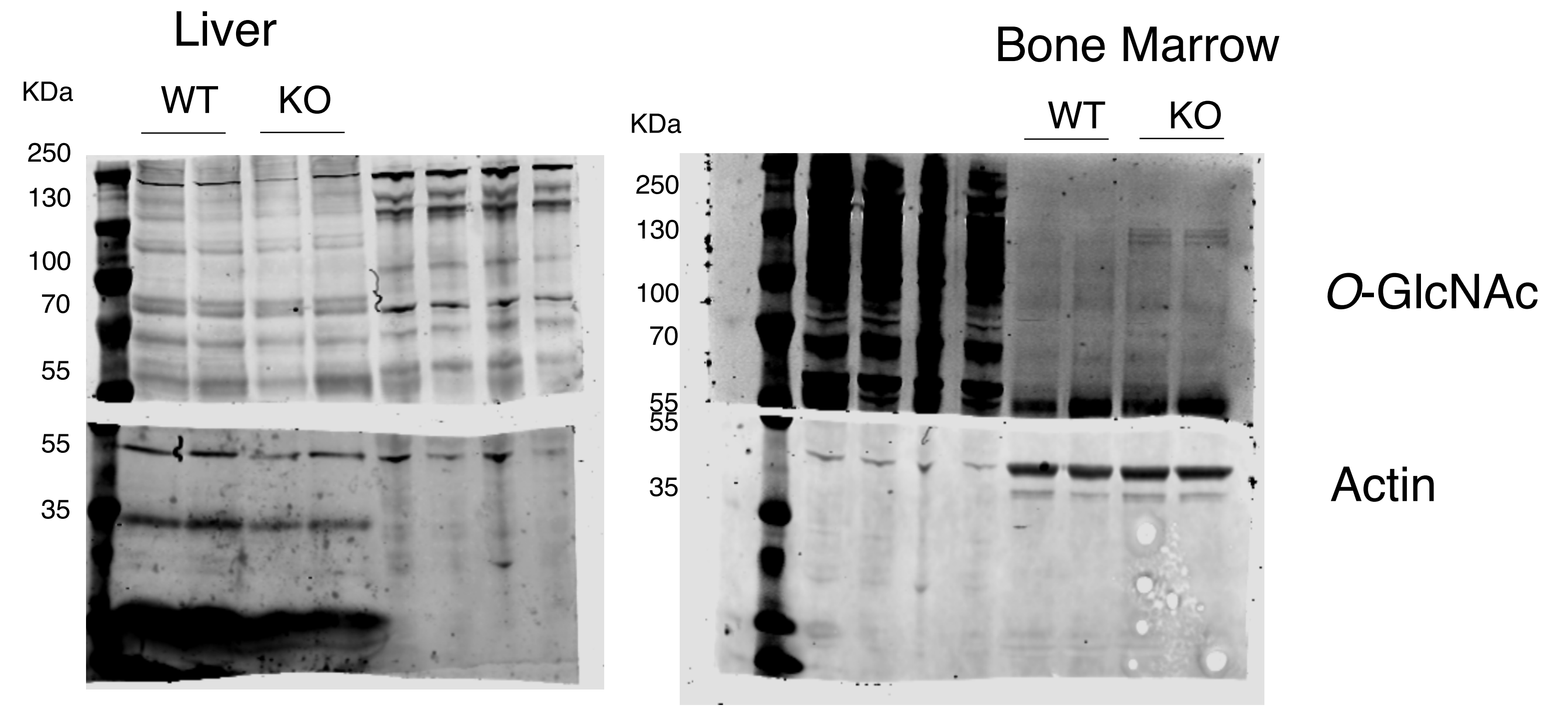

Supplemental Figure S7

Supplemental Table 1. Deregulated genes in Oga deficient LSK cells.

Symbol	log2(fold change)	p_value
Slc1a5	1.444	6.01E-13
4933408J17Rik	1.369	2.57E-06
Tnfsf8	1.343	5.98E-05
LOC100503076	1.336	6.82E-05
4932415G12Rik	1.315	1.06E-06
Ptgir	1.315	3.26E-04
Slamf9	1.209	4.55E-03
Gm5547	1.162	8.08E-03
Tnni1	1.147	1.40E-02
Phlda3	1.039	1.40E-02
Bcam	1.039	2.62E-02
Rasgrf2	1.034	3.42E-02
E330018D03Rik	-5.204	6.97E-92
D19Ertd409e	-4.465	1.01E-129
Fgf3	-3.147	1.24E-25
Mgea5	-3.099	1.04E-132
Mid1	-2.371	1.40E-18
Rbm44	-2.027	1.55E-09
Slc5a9	-2.017	1.96E-09
Tdrkh	-1.813	1.69E-14
Sgip1	-1.634	3.41E-06
Elavl4	-1.547	1.56E-05
Gbp11	-1.5	7.57E-05
E430024I08Rik	-1.371	5.12E-07
Arhgef25	-1.254	3.97E-03
Vldlr	-1.247	6.45E-07
Zfp37	-1.246	3.21E-03
Rnf208	-1.211	6.79E-03
Clip3	-1.194	6.90E-03
1200009P19Rik	-1.174	8.08E-03
Catsperg1	-1.158	8.08E-03
Zscan18	-1.149	2.29E-03
Gng7	-1.131	1.65E-02
Zmym6	-1.113	1.71E-06
AI414108	-1.112	1.64E-02
1810073O08Rik	-1.108	6.79E-03
4930447C04Rik	-1.105	2.02E-02
Creb5	-1.104	2.03E-02
Zfp583	-1.097	1.48E-02
Zfp661	-1.095	3.53E-03
Megf8	-1.072	1.38E-05
Ptpdc1	-1.063	8.54E-04
Mlph	-1.06	1.32E-02
Zfp354b	-1.056	2.86E-02
Dnalc1	-1.046	6.79E-03
Mrc2	-1.044	3.37E-02
Gm15247	-1.032	1.63E-02
Zmynd15	-1.032	3.37E-02
Bpil2	-1.03	7.01E-03
Acad10	-1.027	2.65E-03
Zfp354a	-1.022	1.69E-02
1700029J07Rik	-1.019	3.34E-02
Gstk1	-1.002	2.33E-02

Supplemental Table 2. Primer sequences.

Primer name	Sequence (5'-3')
Slc1a5 F	cccctcctgaaacagtacca
Slc1a5 R	agcctctccaggaaggagac
Fgf3F	tttgtctaccagggccactc
Fgf3R	aaggcatgcaagttcttggt
Eef2F	aggccgccatgggtattaag
Eef2R	aaggcatagaagcggccttt
Rpl38F	gaggatcgctgtgcgga
Rpl38R	tgacagacttggcatccttcc
Rplp0F	atcactgccacccagaacac
Rplp0R	atccacgacggacacattgg
Oga7F	cccccccggagaagataa
Oga7R	caccgcctcctcctccgacaaatc
oIMR9266	agatgccaggacatcaggaacctg
oIMR9267	atcagccacaccagacacagagatc
